# Recent progress of endoplasmic reticulum stress in the mechanism of atherosclerosis

**DOI:** 10.3389/fcvm.2024.1413441

**Published:** 2024-07-12

**Authors:** Lin Ni, Luqun Yang, Yuanyuan Lin

**Affiliations:** Third Hospital of Shanxi Medical University, Shanxi Bethune Hospital, Shanxi Academy of Medical Sciences, Tongji Shanxi Hospital, Taiyuan, China

**Keywords:** endoplasmic reticulum stress, atherosclerosis, inflammatory, cell apoptosis, mitochondrial dysfunction, oxidative stress, autophagy

## Abstract

The research progress of endoplasmic reticulum (ER) stress in atherosclerosis (AS) is of great concern. The ER, a critical cellular organelle, plays a role in important biological processes including protein synthesis, folding, and modification. Various pathological factors may cause ER stress, and sustained or excessive ER stress triggers the unfolded protein response, ultimately resulting in apoptosis and disease. Recently, researchers have discovered the importance of ER stress in the onset and advancement of AS. ER stress contributes to the occurrence of AS through different pathways such as apoptosis, inflammatory response, oxidative stress, and autophagy. Therefore, this review focuses on the mechanisms of ER stress in the development of AS and related therapeutic targets, which will contribute to a deeper understanding of the disease's pathogenesis and provide novel strategies for preventing and treating AS.

## 1 Introduction

Atherosclerosis (AS) serves as the primary pathological foundation of cardiovascular disease (1), encompassing complex metabolic and signaling pathways. The development of AS entails pathological mechanisms including lipid accumulation, foam cell formation, inflammation, endothelial dysfunction, and localized oxidative stress. Risk factors such as dyslipidemia, hyperglycemia, hyperhomocysteinemia (HHcy), and other metabolic disorders induced by genetic factors, dietary choices, and obesity expedite the advancement of AS (2). These pathological processes may involve signaling pathway activation such as inflammatory responses and apoptosis, leading to cellular dysfunction and affecting atherosclerotic plaque initiation and stabilization (3, 4). Emerging research indicates that endoplasmic reticulum (ER) stress plays a role in the pathogenesis of AS through various complex pathways and in a synergistic interplay of multiple risk factors (5). Physiological or pathological triggers, such as oxidative stress, disrupted protein glycosylation, ischemia, hypoxia, and pathogens or their components such as endotoxins, calcium imbalance, and abnormal protein folding, cause misfolded or unfolded proteins to accumulate in the ER, disrupting ER homeostasis, a process called ER stress (6, 7).

When cells encounter such stressors, the ER activates the unfolded protein response (UPR), which initiates downstream signaling pathways. UPR is a cellular stress response that originates from the ER. Accumulation of unfolded or misfolded proteins in the ER lumen causes the 78 kDa glucose-regulated protein (BIP/GRP78) to dissociate from three sensors, namely, protein kinase RNA-like endoplasmic reticulum kinase (PERK), inositol-requiring enzyme 1 (IRE1), and activating transcription factor 6 (ATF6), thereby initiating a UPR signaling cascade (8). During the initial phases of ER stress, PERK is stimulated by trans-autophosphorylation and oligomerization. Consequently, it phosphorylates the eukaryotic initiation factor 2α (eIF2α) within eukaryotic cells, thereby reducing protein overload and subsequently attenuating ER stress translation. EIF2α's phosphorylation modulates activating transcription factor 4 (ATF4) mRNA translation, which regulates autophagy, apoptosis, amino acid metabolism, and antioxidant gene transcription (9). Activating the kinase/endoribonuclease activity of IRE1 is achieved through its dimerization and trans-autophosphorylation. This process regulates X-box binding protein 1 (XBP1)-specific mRNAs, resulting in the production of XBP1s, which can convert to active XBP1 proteins (10). During ER stress, ATF6 is translocated to the Golgi by interacting with the coat protein II complex. There, it is treated by the first-site protease and the second-site protease, releasing its cytoplasmic domain fragment (11). This fragment stimulates the upregulation of genes associated with adaptive stress responses. If the UPR cannot maintain normal ER function, ongoing ER stress leads to ER-associated degradation, oxidative stress, autophagy, mitochondrial dysfunction, inflammation, and apoptosis (12). Previous evidence has shown that the activation of the inflammatory reaction, apoptosis, mitochondrial dysfunction, oxidative stress, autophagy, and other mechanistic pathways mediated by ER stress promote AS, exacerbate metabolic disorders and cellular dysfunction in AS, and further affect plaque formation and stability. Therefore, this paper further reviews the pathways of ER stress signaling pathways in AS related to inflammatory response, apoptosis, mitochondrial dysfunction, oxidative stress, autophagy, and relevant therapeutic targets for AS. In this way, we can further deepen our understanding of the pathogenesis of AS while focusing on the relevant roles of ER stress in AS, providing potential new ways of preventing and treating AS.

## 2 ER stress mediates the inflammatory response in AS

Current research points to that ER stress is linked to inflammatory pathways by multiple mechanisms and plays an important role in atherosclerotic cardiovascular disease (13). Previous evidence suggests that the ER stress proteins are expressed in endothelial cells (ECs) within aortic regions prone to AS in the early stages of AS. Inflammatory signaling pathways also induce early endothelial dysfunction (14). ER stress is a crucial mechanism regulating plaque progression. XBP1, which is a molecule downstream of IRE1 and ATF6, plays a significant role in multiple aspects of AS progression, including macrophage apoptosis, foam cell formation, and the production of inflammatory factors (15). Additionally, it is noted that in advanced stages of AS, a substantial quantity of deceased macrophages and vascular smooth muscle cells (VSMCs) is found within the fibrous cap and disrupted plaques, accompanied by the activation of C/EBP-homologous protein (CHOP) and GRP78. It is noteworthy that the stimulation of inflammatory pathways may result in further exacerbation of plaque rupture (16).

**Figure 1:**
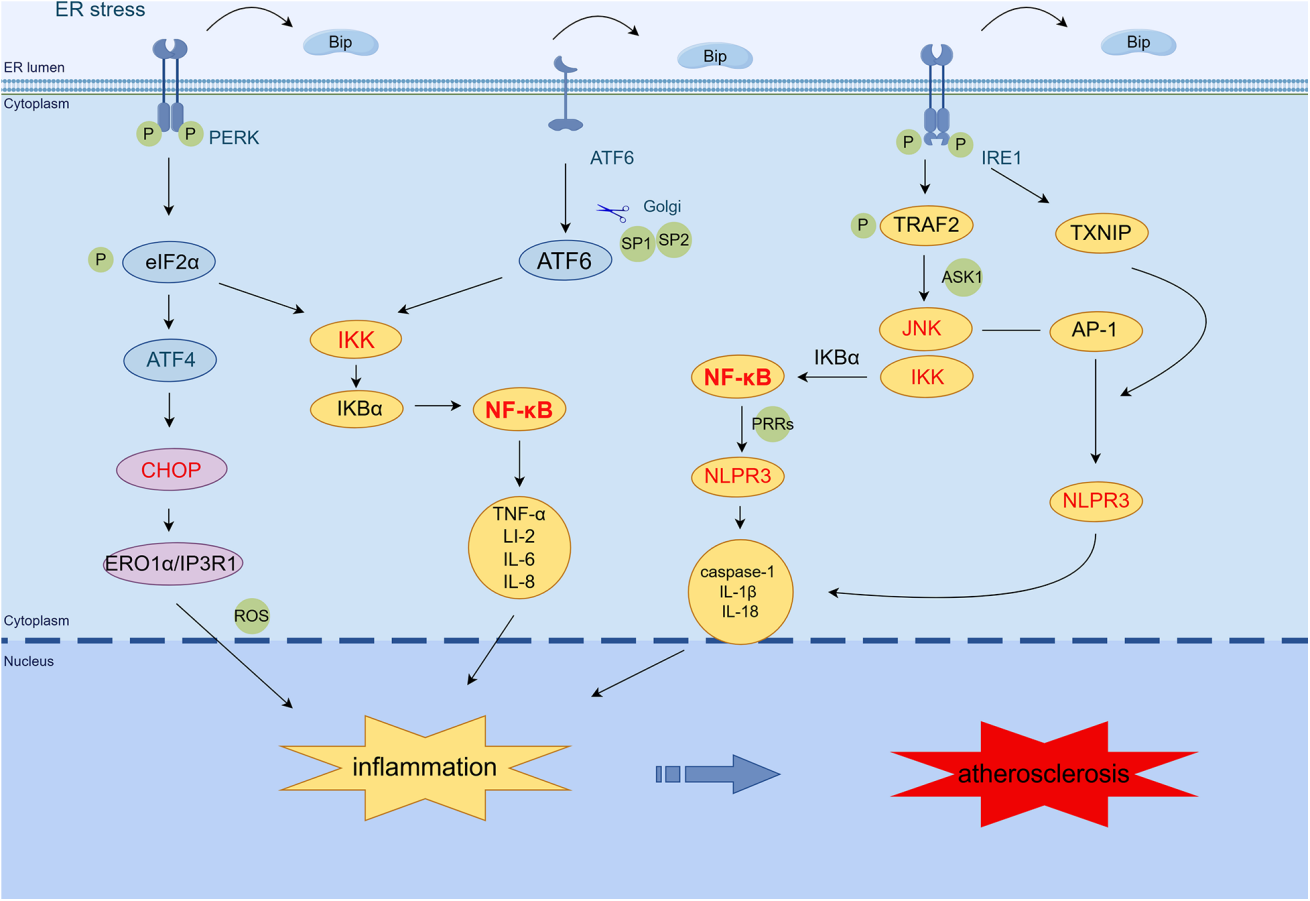
ER stress mediates the inflammatory response in AS. PERK induces NF-κB activation primarily through translational attenuation of free IκBα mediated by phosphorylation of eIF2α. The IRE1α·TRAF2·IKK complex induces IκBα degradation, NF-κB activation, and NLRP3 inflammasome activation. In addition, activated JNK promotes the formation of NLRP3 inflammasome by phosphorylating the transcription factor AP-1 and induces the expression of caspase-1, IL-1β, IL-18, and other inflammatory genes. IRE1α increases the expression of TXNIP protein, which activates the NLRP3 inflammasome, leading to caspase-1 dissociation and IL-1β secretion. The ATF6 branch activates the expression of NF-κB and inflammatory genes such as TNF-*α*, IL-2, IL-6, and IL-8 through the transient phosphorylation of Akt. PERK/ATF4/CHOP transcription activates ERO1α, increases ROS production, and induces inflammation. (By Figdraw.) TRAF2, tumor necrosis factor (TNF) receptor-associated factor-2; JNK, c-Jun N-terminal kinase; ASK1, apoptotic signaling kinase-1; IKK, IκB kinase; IκB, inhibitor of NF-κB; NF-κB, nuclear factor-κB; AP-1, activator protein-1; NLRP3, NOD-, LRR-, and pyrin domain-containing protein 3; CHOP, C/EBP-homologous protein; ERO1α, endoplasmic reticulum oxidoreductase 1 alpha; IP3R1, inositol 1,4,5-trisphosphate receptor type 1; PRRs, pattern recognition receptors; TXNIP, thioredoxin-interacting protein; ROS, reactive oxygen species.

ER stress is involved in the inflammatory response in AS through nuclear factor-κB (NF-κB), c-Jun N-terminal kinase (JNK) signaling pathway, activator protein-1 (AP-1), reactive oxygen species (ROS) generation, and NOD-, LRR-, and pyrin domain-containing protein 3 (NLRP3) inflammasome (17). The NF-κB-IKK pathway is critical to induce inflammatory mediators. PERK, ATF6, and IRE1 can activate the NF-κB pathway and trigger particular inflammatory reactions (18). The PERK/eIF2α pathway induces the activation of NF-κB by mediating the translation attenuation of free IκBα, thereby promoting the expression of tumor necrosis factor-α (TNF-α), IL-2, IL-6, IL-8, and other inflammatory genes (19). IRE1α kinase activation can recruit the adaptor protein TNF receptor-associated factor-2 (TRAF2), thereby activating the JNK-IKK signaling pathway. The IRE1α–TRAF2–IKK complex induces IκBα degradation, NF-κB activation, and NLRP3 inflammasome activation (20, 21). In addition, activated JNK promotes the formation of NLRP3 inflammatory vesicles and induces inflammatory gene expression, such as caspase-1, IL-1β, and IL-18, by phosphorylating transcription factor AP-1 (22, 23). IRE1α hyperactivity stabilizes thioredoxin-interacting protein (TXNIP) mRNA by decreasing microRNA-17 levels. This usually increases TXNIP protein expression, NLRP3 inflammasome activation, caspase-1 dissociation, and IL-1β secretion (24, 25). The ATF6 pathway activates NF-κB by transiently phosphorylating Akt and also triggers the expression of inflammatory genes such as TNF-α, IL-2, IL-6, and IL-8 (26). The PERK/ATF4/CHOP transcription pathway activates endoplasmic reticulum oxidoreductase 1 alpha (ERO1α), increases ROS production, and induces inflammation (27). Taken together, the ER stress pathway promotes inflammatory responses via TRAF2/JNK/NF-κB, JNK/AP-1/NLRP3, CHOP/ERO1/ROS, and NLRP3 inflammatory vesicles (Figure 1).

In addition, the NLRP3 inflammasome signaling cascade is split into three stages: transcription initiation, posttranslational modification, and activation (28). In the transcription initiation phase, damage-related molecular patterns or pathogen-associated molecular patterns linked to pattern recognition receptors induce the initial pathway. This signal then elicits the NF-κB/MAPK signaling pathway, upregulating NLRP3, pro-IL-1β, and pro-IL-18. Within the NLRP3 receptor, JNK-mediated ASC phosphorylation and NLRP3 ubiquitination mediated by the linear ubiquitin chain assembly complex are critical to posttranslational regulation. When ion fluxes (K^+^ efflux, Ca^2+^ influx, and Cl^−^ efflux), mitochondrial damage, ROS generation, lysosomal rupture, histone B release, and ER stress occur in the cytoplasm, the activation initiates the assembly of the NLRP3 inflammatory vesicle complex, thereby promoting inflammation (29–31).

The inflammatory response induced by ER stress promotes AS progression. It was found that NF-κB stimulation produces an increase in inflammatory mediators (e.g., IL-6 and TNF-α), which further induces endothelial dysfunction (14). Endothelial dysfunction is an initial factor in the formation of early atherosclerotic lesions, and proliferative ECs generate substantial NO and exacerbate the inflammatory reaction in AS, which leads to plaque formation (21). Current research shows that multiple risk factors in AS induce inflammatory responses in vascular endothelial cells (VECs). Among these factors, oxidized low-density lipoprotein (ox-LDL) promotes the inflammatory response of NLRP3 via ROS mechanisms, activates caspase-1, and induces VEC stress (32). Nicotine stimulates the NLRP3 inflammasome and promotes inflammatory response and apoptosis in VECs, thereby accelerating the development of AS (33). During the initial stages of AS, macrophage-derived NLRP3 inflammasomes participate in the inflammatory anti-injury response and facilitate plaque stabilization (34). VSMC phenotypic switching mediated by NF-κB enhances the synthesis and reduces the contractility of VSMCs, which is related to extracellular matrix accumulation and plaque progression during AS development (35, 36). NF-κB is also involved in the production of adhesion molecules in the endothelium, including E-selectin, vascular cell adhesion molecule-1, and intercellular adhesion molecule-1, which promote monocyte recruitment (37). Phosphorylation of scavenger receptor type A (SR-A), dependent on JNK2, facilitates lipid uptake by macrophages, thereby modulating the formation of foam cells, a critical stage in atherogenesis (38). IL-1β and other members of the IL-1 cytokine family are important mediators of vascular and systemic inflammation that contribute to atherogenesis. The NLRP3 inflammasome, which is an innate immune signaling complex, serves as a vital mediator for the production of IL-1 family cytokines in AS. NLRP3 can be activated upon exposure to many endogenous danger signals, including ox-LDL and cholesterol crystals, which abound in atheroma lesions (39). Thus, ER stress-induced activation of NF-κB, JNK, NLRP3, inflammatory vesicles, and inflammatory factors participate in the vascular inflammatory response which promotes the onset of AS.

In the late stages of AS, the NLRP3 inflammasome induces premature macrophage death and massive lipid release, increasing plaque vulnerability (34). Studies have demonstrated that NLRP3, ASC, caspase-1, IL-1β, and IL-18 levels are higher in unstable plaques in comparison to stable plaques (40). ER stress-induced activation of the NLRP3 inflammasome is regulated by the receptor-interacting protein 1 (RIP1) kinase. Pharmacological inhibitors of RIP1 phosphorylation and RIP1 activity reduce caspase-1 cleavage and IL-1β secretion mediated by ER stress in macrophages (41). Therefore, blocking NLRP3 signaling reduces pro-inflammatory cytokine production in apolipoprotein E (ApoE) knockout mice and promotes stable plaque through a decrease in macrophages, lipids, and upregulation of SMCs and collagen (42). Specific inhibition of NLRP3 by MCC950 prevents the development of AS through attenuation of inflammation and pyroptosis in macrophages (43).

## 3 ER stress mediates apoptosis in AS

### 3.1 Endothelial cell apoptosis

**Figure 2:**
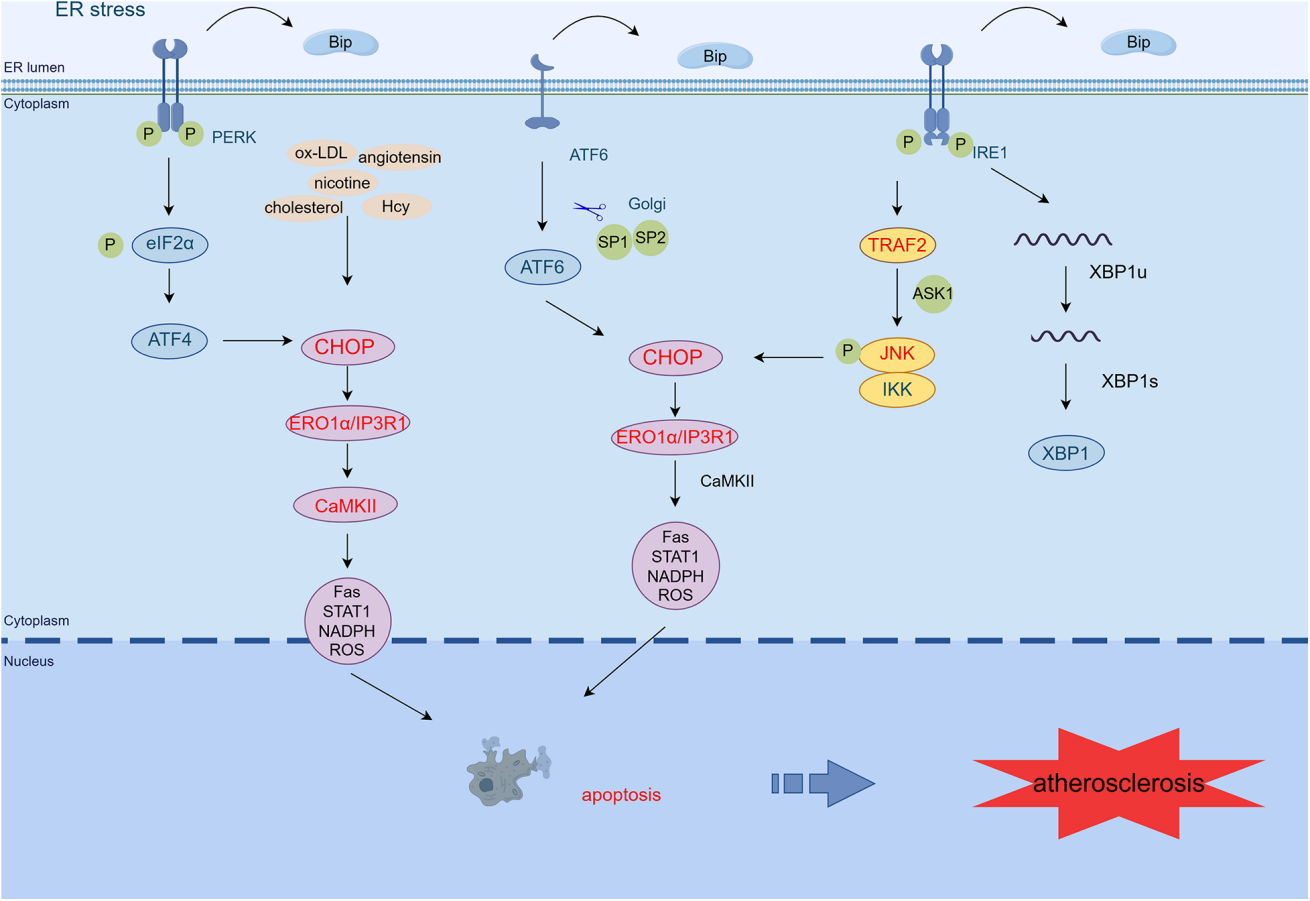
ER stress mediates apoptosis in AS. PERK/eIF2α/ATF4, IRE1α/JNK/MAPK, and ATF6 signaling pathways activate CHOP, which transcribes ERO1α, enhances IP3R1 calcium channel activity, and activates CaMKII. CaMKII activates many pro-apoptotic pathways (Fas, STAT1, NADPH, ROS), leading to macrophage apoptosis. In addition, atherosclerosis-related stressors in endothelial cells (such as oxidized low-density lipoprotein, homocysteine, nicotine, and angiotensin) can activate PERK, IRE1, ATF6, and CHOP/ERO1α signaling pathways and promote cell apoptosis and vascular endothelial dysfunction. Exposure of smooth muscle cells to cholesterol activates the PERK/eIF2α/ATF4 pathway, promoting apoptosis (By Figdraw.) TRAF2, tumor necrosis factor (TNF) receptor-associated factor-2; JNK, c-Jun N-terminal kinase; ASK1, apoptotic signaling kinase-1; IKK, IκB kinase; CHOP, C/EBP-homologous protein; ERO1α, endoplasmic reticulum oxidoreductase 1 alpha; IP3R1, inositol 1,4,5-trisphosphate receptor type 1; CaMKII, calcium/calmodulin-dependent protein kinase II; Hcy, homocysteine.

ER stress is present at all stages of AS, even before free cholesterol accumulation (5). Endothelial dysfunction in areas prone to arterial vascular disease is essential for the pathobiology of atherosclerotic cardiovascular disease (44). In recent years, low shear stress disturbance has emerged as a primary atherosclerotic determinant of EC dysfunction. It may be a direct inducer of ER stress in ECs, which is critically important for AS progression (45). Canham et al. (46) found increased eva-1 homolog A mRNA and protein expression in areas of disturbed EC blood flow, promoting EC dysfunction and inflammatory marker expression through modulating autophagic fluxes in ECs exposed to disturbed flow (Figure 2).

During AS, the oxidation, glycosylation, or phospholipid digestion of LDL, which may disrupt ER calcium metabolism, induces the UPR and oxidative stress in the endothelium by inhibiting sarcoplasmic/ER Ca^2+^-ATPase (47). Among these processes, ox-LDL induces VEC apoptosis primarily via the PERK/eIF2α/CHOP pathway (48). Moreover, ox-LDL inhibits the proliferation of ECs through the ER stress/ASK1 axis and induces apoptosis, ROS production, and inflammatory responses (49). Knockdown of ADAMDEC1 (a disintegrin and metalloproteinase domain-like protein decysin 1) ameliorates ox-LDL-induced EC injury and AS progression (50).

Moreover, the accumulation of the active dicarbonyl metabolite methylglyoxal (MG) is also an important factor in EC dysfunction. In ECs exposed to high glucose levels, the increased formation of MG, decreased glyoxalase 1 activity, and the synergistic combination of these two metabolic changes result in “dicarbonyl stress,” activating URP, and further pro-inflammatory, pro-atherogenic, and pro-thrombotic (51). In human umbilical vein ECs, Hcy-induced ER stress has been shown to induce definite modifications in gene expression and programmed cell death (52). Recently, it has been shown that Hcy induces endothelial dysfunction by inhibiting calcium-activated potassium channels, mediated by ER stress (53). Angiotensin 1–7 prevents angiotensin II-mediated ER stress and endothelial dysfunction by Mas receptors (54). Angiotensin 1–9 inhibits apoptosis in EC through suppression of CNPY2/PERK-mediated calcium/calmodulin-dependent protein kinase (CaMK) II/Drp1-dependent mitochondrial fission and eIF2α/CHOP signaling (55). Hydrogen sulfide inhibits endothelin-1 production stimulated by angiotensin II and following cytotoxic-mediated ER stress in ECs through NF-κB (56). Other relevant stressors such as IRE1α and PERK phosphorylation and increased expression of ATF6 and GRP78 in nicotine-treated ECs aggravated glucose-induced ER stress and apoptosis (8). Thus, AS-associated stressors induce endothelial dysfunction via the ER stress pathway, which further promotes AS formation.

### 3.2 Macrophage apoptosis

Macrophage apoptosis, induced by ER stress, plays an essential role in the formation of necrotic and inflamed cores. This process significantly contributes to plaque destabilization, thereby establishing a foundation for potential plaque rupture (3, 57).

Studies have shown that atherogenic lipids, such as oxidized phospholipids, ox-LDL, saturated fatty acids, and lipoprotein (a), upregulate scavenger receptors such as SR-A, CD36, and lectin-like ox-LDL receptor-1 by activating ER stress (58–60). CD36 activates Src family kinases, MAP kinases, and Vav family guanine nucleotide exchange factors, resulting in ligand formation *in vivo* and the formation of foam cells (60). SR-A1 and CD36 activation leads to increased free cholesterol deposition in the ER, induces ER stress, accelerates macrophage apoptosis, and causes atherosclerotic plaque necrosis and instability (61, 62). Toll-like receptor 4 mediates macrophage cholesterol accumulation and activation of ER stress via IRE1 and ATF6 pathways (63). Reduced expression of the genes for ATP-binding cassette transporter G1 (ABCG1) and ATP-binding cassette transporter A1 (ABCA1) is another significant characteristic of foam cell formation and the downregulation of class B type I scavenger receptor upon activation of the UPR pathway, thereby interfering with cholesterol efflux (64). Methyl dihydrogen phosphate can downregulate Cav1.2 channels to prevent extracellular Ca^2+^ influx, reduce intracellular Ca^2+^ levels, inhibit calcium-activated calpain activity, and reduce the strength of the calpain-ABCA1 interaction, increasing ABCA1 stability and stimulating cellular cholesterol efflux (65).

Research conducted on cultured macrophages has found that CHOP expression increases as ER stress develops, under atherosclerotic conditions. It is a commonly used pathway for ER stress-mediated macrophage apoptosis (66). A complete apoptotic response requires initiation of the upstream CHOP pathway in addition to the downstream apoptotic Fas and mitochondrial pathways (67, 68). CHOP induces ERO1, leading to ER lumen overoxidation. This process activates the inositol 1,4,5-trisphosphate receptor type 1 (IP3R1) and forms disulfide bonds in the luminal loop of IP3R1. This process ultimately enhances the activity of the IP3R1 calcium channel, leading to an increase in calcium release (69). Sustained increases in cytoplasmic calcium activate CaMKII, thereby activating multiple pro-apoptotic pathways, involving Fas, mitochondrial apoptotic factors, signal transducer and trigger of transcription 1, and NADPH-mediated ROS (70, 71). In addition, pathways such as IRE1α/JNK/MAPK, ATF6, and PERK/eIF4α/ATF4 can all activate CHOP, thereby leading to apoptosis (71, 72).

### 3.3 Smooth muscle cell apoptosis

The protection of VSMCs from apoptosis is a latent critical therapeutic target for stabilizing atherosclerotic plaques. ER stress-induced apoptosis in VSMCs leads to thinning of the protective collagen cap, which may contribute to the transition from stable to fragile forms of advanced atherosclerotic plaques (73). Furthermore, apoptosis of VSMCs accelerates AS, promotes plaque calcification, and leads to medial degeneration. It also prevents dilated remodeling and promotes stenosis within AS (74). Exposure to cholesterol activates UPR pathways, increasing KLF4 (Krüppel-like factor 4) expression via the PERK/eIF2α/ATF4 pathway (75). It was found that targeted knockdown of KLF4 reduced Lgals3 in VSMCs and enhanced contractile markers in VSMCs, thereby promoting plaque stabilization (76, 77). VSMC phenotypic switching also is essential in AS. Knockdown of Hcy-inducible ER protein attenuates AS mediated by HHcy through the inhibition of VSMC phenotypic switching (78). Intimal hyperplasia is a common complication in restenosis and atherosclerotic plaques. Proliferation, migration, and inflammatory phenotype switching of VSMCs are key factors underlying intimal hyperplasia (79). Knockdown of the SMYD3-PARP16 signaling axis inhibits ER stress, subsequently reducing VSMC proliferation and migration, which ultimately decreases intimal hyperplasia (80).

## 4 ER stress mediates mitochondrial dysfunction and oxidative stress in AS

ER stress and its induced mitochondrial dysfunction trigger progressive cell death, cellular stress, and ROS accumulation. Increased mitochondrial ROS production, accumulation of mitochondrial DNA damage, and progressive dysfunction of the respiratory chain are all associated with AS (81, 82).

Alterations in ER redox homeostasis are adequate to induce ER stress, which can subsequently lead to ROS production in both mitochondria and ER. Excess ROS-induced oxidative stress is now recognized as an indispensable part of the progression of AS (83). The oxidative stress promotes critical lesions in AS including oxidative modification of lipoproteins and phospholipids, EC activation, and macrophage infiltration (84). Mitochondria, which form tight complexes with the ER via ER-associated mitochondrial membranes (MAMs), are a primary source of ROS (85). Under ER stress, the accumulation of ROS can disrupt the transfer of Ca^2+^ and protein folding. ER stress worsens mitochondrial dysfunction, resulting in a significant reduction in mitochondrial membrane potential, impairment of oxidative phosphorylation, and additional pathological situations (86). ER stress induces the production of ROS in the ER and mitochondria. Under ER stress, abundant activated JNK associates with the MAM connexin Sab, resulting in the release of ROS alongside mitochondria (87). Prevention of protein translation through the PERK/eIF2α/ATF4 pathway causes increased production of ROS and reduced antioxidants (88). Moreover, NADPH oxidases, particularly NOX2 and NOX4, increase the generation of ROS during ER stress (89). Activation of NOX4 occurs primarily through the PERK/eIF2α/ATF4 and IRE1/XBP1 pathways. Additionally, ROS can enhance inflammasome formation on MAMs, inducing IL-1β and IL-18 production, and downstream inflammatory responses (27). Similarly, the accumulation of ROS induced by TNF-α causes ER stress (90).

In addition to being a significant pro-apoptotic factor in response to unfolded proteins, CHOP also induces oxidative stress through various mechanisms. In mammalian cells, CHOP transcriptionally activates ERO1α, which increases ROS production during ER stress. CaMKII also triggers the activation of the NOX subunit NOX2, resulting in oxidative stress. During ER stress, oxidative stress further induces CHOP induction dependent on PKR and creates a positive feedforward cycle. CHOP enhances cell death via the restoration of global mRNA translation, which results in protein misfolding and induces mitochondria-dependent oxidative stress (91).

Mitochondrial dysfunction enhances endothelial oxidative stress, perturbs endothelial nitric oxide metabolism and cytosolic calcium cycling, triggers an inflammatory response, accelerates senescence, inhibits proliferation, and promotes apoptosis. It is closely associated with endothelial dysfunction (92). Cholesterol efflux is regulated by ATP-dependent transporter proteins, namely, ABCA1 and ABCG1. However, when ATP synthesis is compromised—a condition often associated with mitochondrial dysfunction—it disrupts lipid metabolism and consequently leads to the inhibition of cholesterol efflux (93). Inhibiting mitochondrial oxidative stress signaling in macrophages by overexpressing mitochondrial catalase has been shown to reduce lesion area, inflammatory signaling, and immune cell infiltration into the aortic root of Western-fed Ldlr^−/−^ mice (94). In addition, degradation of endothelial nitric oxide synthase (eNOS) induced by ROS-mediated oxidative stress leads to decreased NO formation and promotes the development of endothelial dysfunction in early AS. Dysfunctional eNOS uncoupling generates more ROS, further impairing endothelial function and promoting the development of AS (95). Excessive ROS can induce endothelial dysfunction, vascular inflammation, and accumulation of ox-LDL in the arterial wall. These processes contribute to the initial formation of lesions and the maturation of advanced plaques, which may eventually progress to plaque rupture (96). ROS also stimulates the release of matrix metalloproteinases, which degrade the fibrous wall of atherosclerotic plaques and the basement membrane of ECs, thereby impacting plaque stability (97). NOX2ds-tat, an inhibitor of NOX2 oxidase, reduces atherosclerotic plaque formation by inhibiting oxidative stress and angiogenic mediator production and reducing plaque neovascularization associated with vulnerability to atherosclerotic lesions (98). Thus, ER stress-induced mitochondrial dysfunction and oxidative stress contribute to the progression of AS.

## 5 ER stress mediates autophagy in AS

**Figure 3:**
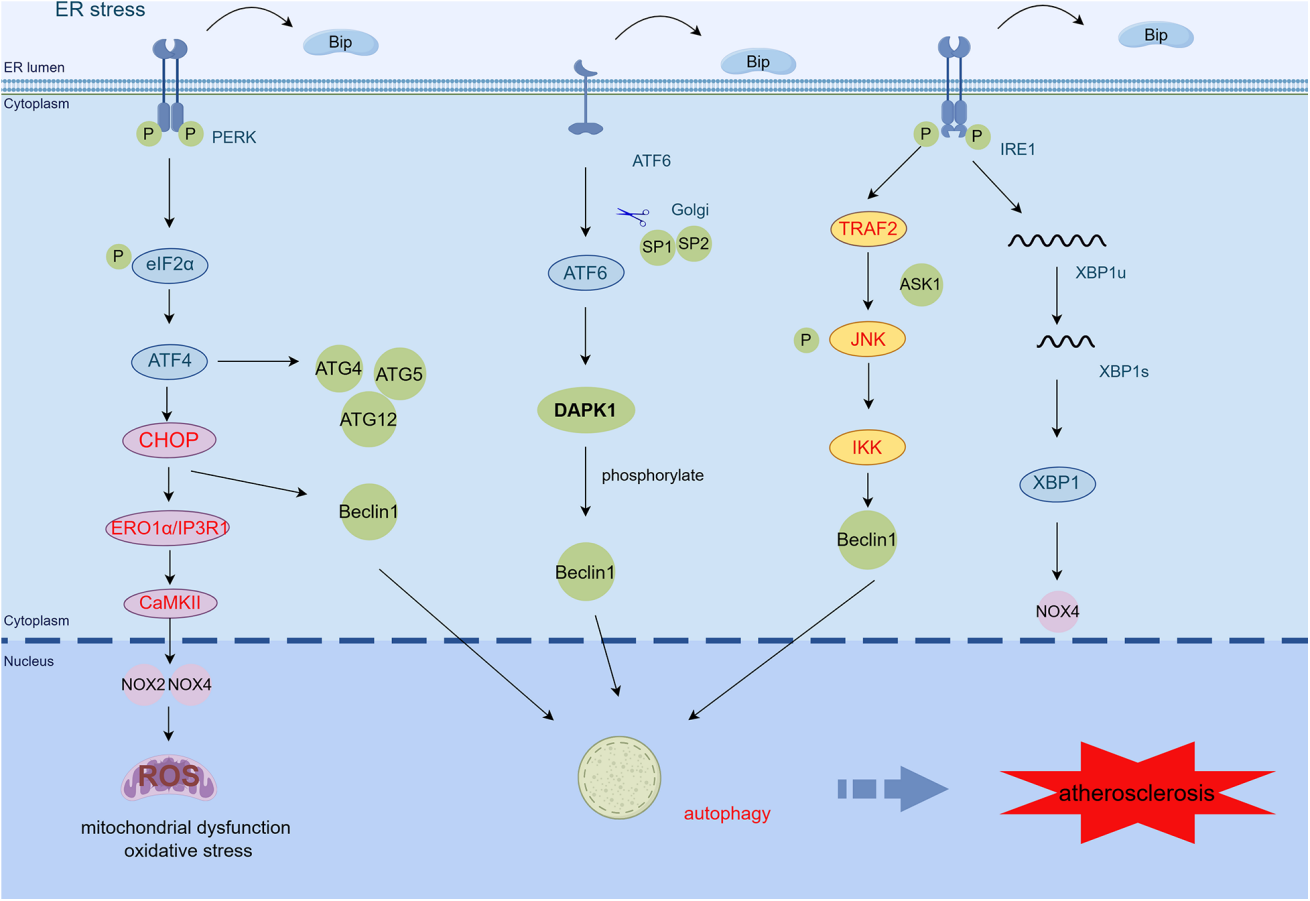
ER stress mediates mitochondrial dysfunction, oxidative stress, and autophagy in AS. CHOP activates the ERO1α/CaMKII signaling pathway by transcription and increases the production of NOX2, NOX4, and ROS, thereby inducing oxidative stress and mitochondrial dysfunction. PERK/eIF2α/ATF4 activates upregulation of autophagy genes such as ATG4, ATG5, and ATG12. The PERK/eIF2α/ATF4 and IRE1α/JNK signaling pathways also induce autophagy by increasing free Beclin1. ATF6 regulates autophagy by upregulating the expression of DAPK1, which phosphorylates Beclin1 (By Figdraw.) TRAF2, tumor necrosis factor (TNF) receptor-associated factor-2; JNK, c-Jun N-terminal kinase; ASK1, apoptotic signaling kinase-1; IKK, IκB kinase; CHOP, C/EBP-homologous protein; ERO1α, endoplasmic reticulum oxidoreductase 1 alpha; IP3R1, inositol 1,4,5-trisphosphate receptor type 1; CaMKII, calcium/calmodulin-dependent protein kinase II; DAPK1, death-associated protein kinase 1; ROS, reactive oxygen species.

Autophagy genes (e.g., ATG4, ATG5, and ATG12) are increased by PERK-phosphorylated eIF2α activation when ER stress occurs (99). Additionally, eIF2α-induced CHOP reduces Beclin1-Bcl2 complex formation, upregulates free Beclin1, and increases ER autophagy (100). CHOP also increases LC3 expression through the localization of more autophagosomes to damaged ER regions and the subsequent degradation of ER fragments, while decreasing phosphorylation of mTOR (100). Inhibition of mTOR can increase macrophage cholesterol efflux protein expression by promoting the translocation of transcription factor EB to the nucleus (101). The UPR leads to the activation of IRE1, which complexes with ASK1-TRAF2 and activates the JNK pathway, increasing free Beclin1 and inducing autophagy (100). In addition, the ATF6 pathway causes upregulation of the expression of death-associated protein kinase 1 (DAPK1). DAPK1 mediates autophagy by phosphorylating Beclin1 (102) (Figure 3).

There are many triggers of autophagy in atherosclerotic plaques, such as LDL, ROS, inflammatory cytokines, and TNF-α (103). Low levels of cellular stress, such as oxidative stress, ox-LDL, and ER stress, can stimulate low levels of adaptive autophagy in vascular cells, promoting cell survival through the degradation of injured organelles and proteins, thereby defending the vascular tissue against oxidative stress or inflammation (104). Research has demonstrated that autophagy reduces the area of atherosclerotic plaques and maintains a stable phenotype of plaques. This includes reducing lipid deposition and pro-inflammatory macrophages, while simultaneously increasing anti-inflammatory macrophages, collagen content, and VSMCs. Additionally, it minimizes cell death within the plaque (105). D4F, a primary anti-atherogenic component of high-density lipoprotein (HDL), attenuates CHOP-mediated apoptosis in macrophages exposed to glycated HDL through the promotion of autophagy (106).

Serious oxidative stress or inflammation can provoke excessive autophagy, resulting in autophagy-dependent cell death, reduced collagen synthesis, a thinned fibrous cap, plaque instability, and acute vascular events (107). Recent studies have revealed that defects in autophagy due to specific VSMC knockdown of human antigen R trigger plaque formation and plaque instability (108). Overexpression of sirtuin 6 activates macrophage autophagy, which can inhibit apoptosis, reduce the formation of foam cells, and stabilize atherosclerotic plaques (109). Therefore, early resistance to excessive autophagy is essential to prevent AS and avoid serious cardiovascular complications.

## 6 ER stress therapy for AS-related targets

These micromolecular “chemical chaperones” can mitigate ER protein burden upon stress by the non-selectively stabilization of unfolded proteins and promotion of normal folding. Tauroursodeoxycholic acid (TUDCA) and 4-phenylbutyric acid (4-PBA) are two “chemical partners” authorized for use in humans by the FDA (110). 4-PBA has been approved as an ammonia scavenger for the clinical management of urea cycle disorders (111). It is also utilized in the treatment of other diseases associated with protein misfolding. The current study found that inhibition of ER stress by 4-PBA alleviated endothelial dysfunction and attenuated the effects of ox-LDL on cholesterol efflux, apoptosis, ROS production, and inflammation (49). 4-PBA attenuates atherosclerotic lesion progression through increased circulation of HSP25 in macrophages and ApoE^−/−^ mice (112). In addition, 4-PBA has demonstrated inhibition of the upregulation of CD36, GRP78, and IRE1 phosphorylation in macrophage-rich atherosclerotic lesions (58). Recent research shows that inhibiting ER stress with 4-PBA enhances atherosclerotic plaque stability through upregulation of circadian locomotor output cycles kaput expression (113). TUDCA, a derivative of endogenous bile acids, is used as a hepatoprotective agent in patients with cholestatic liver disease (110). Previous studies have found that TUDCA attenuates atherosclerotic lesion progression in LDL receptor-deficient mice by reducing ER stress (114). TUDCA attenuates atherosclerotic lesions by inhibiting ER stress-mediated apoptosis by reducing calcium efflux, inhibiting cysteine-12, and activating phosphatidylinositol 3-kinase (115). TUDCA further alleviates AS by inhibiting PERK/eIF2α/ATF4 and AIM2 inflammatory vesicles in macrophages to abrogate ox-LDL-induced foam cell formation and enhance cholesterol efflux from macrophages (116). UDCA reduced XBP-1 and CHOP expression, thereby attenuating perturbed flow-induced EC apoptosis, which was induced by inhibiting ER stress in ECs under perturbed flow (45).

Moreover, attenuating the ER stress by inhibiting the UPR signaling cascade is also an important approach for treating AS (8). Furthermore, the blockade of IRE1 kinase-fragile X messenger ribonucleoprotein signaling enhanced cholesterol efflux and exocytosis, thereby reducing AS in mice (117). Sirtuin 1, a deacetylase dependent on NAD, reduces apoptosis mediated by ER stress via inhibition of the PERK/eIF2α signaling pathway (118). Additionally, irisin has been shown to attenuate ox-LDL-induced macrophage apoptosis through inhibition of the PERK/eIF2α/CHOP and ATF6/CHOP signaling pathways (119).

In addition to physiological inhibitors of ER stress aiming at adenosine monophosphate-activated protein kinase (AMPK), regulating ER calcium homeostasis, removing AS-related ER stress inducers, and some natural compounds (8), we can explore new therapeutic directions for AS treatment by targeting ER stress-mediated pathways such as inflammation, apoptosis, mitochondrial dysfunction, oxidative stress, and autophagy. For instance, Bruton's tyrosine kinase knockdown inhibits ox-LDL-mediated activation of NK-κB signaling and suppresses M1 polarization in macrophages. It also inhibits ER stress, oxidative stress, and inflammatory responses in macrophages. Therefore, it emerges as a potential target for the treatment of AS (120). Furthermore, HCP1, a novel coumarin pyrazoline derivative, attenuates ox-LDL-mediated VEC autophagy, reduces apoptosis, and promotes atherosclerotic plaque stabilization in ApoE^−/−^ mice through the Grp94-AMPK-mTORC1 pathway (121). The GLP-1 analog, exendin-4, ameliorates HHcy-induced endothelial dysfunction by activating AMPK and upregulating ERO1α to inhibit the ER stress induced by HHcy and subsequent ROS production (122). Moreover, currently prescribed medications for hypertension, obesity, and diabetes—such as enalapril, valsartan, atorvastatin, and metformin—and natural compounds, such as mangosteen, black tea, and *Panax ginseng*, are recognized for their protective effects on cardiovascular function by inhibiting ER stress (123–126). In summary, targeting the ER stress pathway provides a potential therapeutic strategy for the treatment of AS.

## 7 Conclusion

Increasing research shows that targeting the ER stress and UPR signaling pathways is a new direction in the management of AS. This review highlights the effects of ER stress on the inflammatory response, apoptosis, mitochondrial dysfunction, oxidative stress, and autophagy in AS plaque formation and stability, thus verifying its significant role in AS pathogenesis. However, to further explore and validate the molecular mechanisms and signaling pathways in ER stress-mediated AS, additional experimental studies are necessary, thereby identifying potential new therapeutic targets for AS and related cardiovascular diseases.

## References

[1] GBD 2019 Diseases and Injuries Collaborators. Global burden of 369 diseases and injuries in 204 countries and territories, 1990–2019: a systematic analysis for the global burden of disease study 2019. Lancet. (2020) 396(10258):1204–22. 10.1016/S0140-6736(20)30925-9 33069326 PMC7567026

[2] Weber C Noels H. Atherosclerosis: current pathogenesis and therapeutic options. Nat Med. (2011) 17(11):1410–22. 10.1038/nm.2538 22064431

[3] Tabas I. Macrophage death and defective inflammation resolution in atherosclerosis. Nat Rev Immunol. (2010) 10(1):36–46. 10.1038/nri2675 19960040 PMC2854623

[4] Soehnlein O Libby P. Targeting inflammation in atherosclerosis—from experimental insights to the clinic. Nat Rev Drug Discov. (2021) 20(8):589–610. 10.1038/s41573-021-00198-1 33976384 PMC8112476

[5] Zhou J Lhoták S Hilditch BA Austin RC. Activation of the unfolded protein response occurs at all stages of atherosclerotic lesion development in apolipoprotein E-deficient mice. Circulation. (2005) 111(14):1814–21. 10.1161/01.CIR.0000160864.31351.C1 15809369

[6] Pastor-Cantizano N Ko DK Angelos E Pu Y Brandizzi F. Functional diversification of ER stress responses in Arabidopsis. Trends Biochem Sci. (2020) 45(2):123–36. 10.1016/j.tibs.2019.10.008 31753702 PMC6980780

[7] Lebeaupin C Vallée D Hazari Y Hetz C Chevet E Bailly-Maitre B. Endoplasmic reticulum stress signalling and the pathogenesis of non-alcoholic fatty liver disease. J Hepatol. (2018) 69(4):927–47. 10.1016/j.jhep.2018.06.008 29940269

[8] Yang S Wu M Li X Zhao R Zhao Y Liu L Role of endoplasmic reticulum stress in atherosclerosis and its potential as a therapeutic target. Oxid Med Cell Longevity. (2020) 2020:9270107. 10.1155/2020/9270107 PMC7499294 32963706

[9] Han J Back SH Hur J Lin YH Gildersleeve R Shan J ER-stress-induced transcriptional regulation increases protein synthesis leading to cell death. Nat Cell Biol. (2013) 15(5):481–90. 10.1038/ncb2738 23624402 PMC3692270

[10] Rojas-Rivera D Rodriguez DA Sepulveda D Hetz C. ER Stress sensing mechanism: putting off the brake on UPR transducers. Oncotarget. (2018) 9(28):19461–2. 10.18632/oncotarget.25114 29731957 PMC5929400

[11] Ren J Bi Y Sowers JR Hetz C Zhang Y. Endoplasmic reticulum stress and unfolded protein response in cardiovascular diseases. Nat Rev Cardiol. (2021) 18(7):499–521. 10.1038/s41569-021-00511-w 33619348

[12] Walter P Ron D. The unfolded protein response: from stress pathway to homeostatic regulation. Science. (2011) 334(6059):1081–6. 10.1126/science.1209038 22116877

[13] Hotamisligil GS. Endoplasmic reticulum stress and the inflammatory basis of metabolic disease. Cell. (2010) 140(6):900–17. 10.1016/j.cell.2010.02.034 20303879 PMC2887297

[14] Battson ML Lee DM Gentile CL. Endoplasmic reticulum stress and the development of endothelial dysfunction. Am J Physiol Heart Circ Physiol. (2017) 312(3):H355–67. 10.1152/ajpheart.00437.2016 27923788

[15] Sage AP Nus M Bagchi Chakraborty J Tsiantoulas D Newland SA Finigan AJ X-box binding protein-1 dependent plasma cell responses limit the development of atherosclerosis. Circ Res. (2017) 121(3):270–81. 10.1161/CIRCRESAHA.117.310884 28620068

[16] Wang N Zhang X Ma Z Niu J Ma S Wenjie W Combination of tanshinone IIA and astragaloside IV attenuate atherosclerotic plaque vulnerability in ApoE(−/−) mice by activating PI3 K/AKT signaling and suppressing TRL4/NF-κB signaling. Biomed Pharmacother. (2020) 123:109729. 10.1016/j.biopha.2019.109729 31887543

[17] Reverendo M Mendes A Argüello RJ Gatti E Pierre P. At the crossway of ER-stress and proinflammatory responses. FEBS J. (2019) 286(2):297–310. 10.1111/febs.14391 29360216

[18] Li W Jin K Luo J Xu W Wu Y Zhou J NF-κB and its crosstalk with endoplasmic reticulum stress in atherosclerosis. Front Cardiovasc Med. (2022) 9:988266. 10.3389/fcvm.2022.988266 36204587 PMC9530249

[19] Zhang H Zhao C Wang S Huang Y Wang H Zhao J Anti-dsDNA antibodies induce inflammation via endoplasmic reticulum stress in human mesangial cells. J Transl Med. (2015) 13:178. 10.1186/s12967-015-0536-7 26040555 PMC4467615

[20] Yao F Long LY Deng YZ Feng YY Ying GY Bao WD RACK1 Modulates NF-κB activation by interfering with the interaction between TRAF2 and the IKK complex. Cell Res. (2014) 24(3):359–71. 10.1038/cr.2013.162 24323043 PMC3945882

[21] Tufanli O Telkoparan Akillilar P Acosta-Alvear D Kocaturk B Onat UI Hamid SM Targeting IRE1 with small molecules counteracts progression of atherosclerosis. Proc Natl Acad Sci U S A. (2017) 114(8):E1395–404. 10.1073/pnas.1621188114 28137856 PMC5338400

[22] Shen CH Tung SY Huang WS Lu CC Lee KC Hsieh YY Exploring the effects of tert-butylhydroperoxide induced liver injury using proteomic approach. Toxicology. (2014) 316:61–70. 10.1016/j.tox.2013.12.007 24394546

[23] Stöhr D Jeltsch A Rehm M. TRAIL receptor signaling: from the basics of canonical signal transduction toward its entanglement with ER stress and the unfolded protein response. Int Rev Cell Mol Biol. (2020) 351:57–99. 10.1016/bs.ircmb.2020.02.002 32247582

[24] Talty A Deegan S Ljujic M Mnich K Naicker SD Quandt D Inhibition of IRE1α RNase activity reduces NLRP3 inflammasome assembly and processing of pro-IL1β. Cell Death Dis. (2019) 10(9):622. 10.1038/s41419-019-1847-z 31417078 PMC6695440

[25] Lerner AG Upton JP Praveen PV Ghosh R Nakagawa Y Igbaria A IRE1α Induces thioredoxin-interacting protein to activate the NLRP3 inflammasome and promote programmed cell death under irremediable ER stress. Cell Metab. (2012) 16(2):250–64. 10.1016/j.cmet.2012.07.007 22883233 PMC4014071

[26] Yamazaki H Hiramatsu N Hayakawa K Tagawa Y Okamura M Ogata R Activation of the akt-NF-kappaB pathway by subtilase cytotoxin through the ATF6 branch of the unfolded protein response. J Immunol. (2009) 183(2):1480–7. 10.4049/jimmunol.0900017 19561103 PMC2762936

[27] van Vliet AR Verfaillie T Agostinis P. New functions of mitochondria associated membranes in cellular signaling. Biochim Biophys Acta. (2014) 1843(10):2253–62. 10.1016/j.bbamcr.2014.03.009 24642268

[28] Huang Y Xu W Zhou R. NLRP3 inflammasome activation and cell death. Cell Mol Immunol. (2021) 18(9):2114–27. 10.1038/s41423-021-00740-6 34321623 PMC8429580

[29] Pain P Spinelli F Gherardi G. Mitochondrial cation signalling in the control of inflammatory processes. Int J Mol Sci. (2023) 24(23):16724. 10.3390/ijms242316724 PMC10706693 38069047

[30] Jo EK Kim JK Shin DM Sasakawa C. Molecular mechanisms regulating NLRP3 inflammasome activation. Cell Mol Immunol. (2016) 13(2):148–59. 10.1038/cmi.2015.95 26549800 PMC4786634

[31] Chong WC Shastri MD Peterson GM Patel RP Pathinayake PS Dua K The complex interplay between endoplasmic reticulum stress and the NLRP3 inflammasome: a potential therapeutic target for inflammatory disorders. Clin Transl Immunol. (2021) 10(2):e1247. 10.1002/cti2.1247 PMC7878118 33614031

[32] Yin Y Li X Sha X Xi H Li YF Shao Y Early hyperlipidemia promotes endothelial activation via a caspase-1-sirtuin 1 pathway. Arterioscler Thromb Vasc Biol. (2015) 35(4):804–16. 10.1161/ATVBAHA.115.305282 25705917 PMC4376583

[33] Wu X Zhang H Qi W Zhang Y Li J Li Z Nicotine promotes atherosclerosis via ROS-NLRP3-mediated endothelial cell pyroptosis. Cell Death Dis. (2018) 9(2):171. 10.1038/s41419-017-0257-3 29416034 PMC5833729

[34] Tabas I Lichtman AH. Monocyte-macrophages and T cells in atherosclerosis. Immunity. (2017) 47(4):621–34. 10.1016/j.immuni.2017.09.008 29045897 PMC5747297

[35] Lu QB Wan MY Wang PY Zhang CX Xu DY Liao X Chicoric acid prevents PDGF-BB-induced VSMC dedifferentiation, proliferation and migration by suppressing ROS/NFκB/mTOR/P70S6K signaling cascade. Redox Biol. (2018) 14:656–68. 10.1016/j.redox.2017.11.012 29175753 PMC5716955

[36] Kong P Yu Y Wang L Dou YQ Zhang XH Cui Y Circ-Sirt1 controls NF-κB activation via sequence-specific interaction and enhancement of SIRT1 expression by binding to miR-132/212 in vascular smooth muscle cells. Nucleic Acids Res. (2019) 47(7):3580–93. 10.1093/nar/gkz141 30820544 PMC6468289

[37] Pateras I Giaginis C Tsigris C Patsouris E Theocharis S. NF-κB signaling at the crossroads of inflammation and atherogenesis: searching for new therapeutic links. Expert Opin Ther Targets. (2014) 18(9):1089–101. 10.1517/14728222.2014.938051 25005042

[38] Ricci R Sumara G Sumara I Rozenberg I Kurrer M Akhmedov A Requirement of JNK2 for scavenger receptor A-mediated foam cell formation in atherogenesis. Science. (2004) 306(5701):1558–61. 10.1126/science.1101909 15567863

[39] Grebe A Hoss F Latz E. NLRP3 inflammasome and the IL-1 pathway in atherosclerosis. Circ Res. (2018) 122(12):1722–40. 10.1161/CIRCRESAHA.118.311362 29880500

[40] Shi X Xie WL Kong WW Chen D Qu P. Expression of the NLRP3 inflammasome in carotid atherosclerosis. J Stroke Cerebrovasc Dis. (2015) 24(11):2455–66. 10.1016/j.jstrokecerebrovasdis.2015.03.024 26381780

[41] Tao L Lin H Wen J Sun Q Gao Y Xu X The kinase receptor-interacting protein 1 is required for inflammasome activation induced by endoplasmic reticulum stress. Cell Death Dis. (2018) 9(6):641. 10.1038/s41419-018-0694-7 29844315 PMC5974395

[42] Zheng F Xing S Gong Z Mu W Xing Q. Silence of NLRP3 suppresses atherosclerosis and stabilizes plaques in apolipoprotein E-deficient mice. Mediat Inflamm. (2014) 2014:507208. 10.1155/2014/507208 PMC4066943 24999295

[43] Zeng W Wu D Sun Y Suo Y Yu Q Zeng M The selective NLRP3 inhibitor MCC950 hinders atherosclerosis development by attenuating inflammation and pyroptosis in macrophages. Sci Rep. (2021) 11(1):19305. 10.1038/s41598-021-98437-3 34588488 PMC8481539

[44] Davies PF Civelek M Fang Y Fleming I. The atherosusceptible endothelium: endothelial phenotypes in complex haemodynamic shear stress regions in vivo. Cardiovasc Res. (2013) 99(2):315–27. 10.1093/cvr/cvt101 23619421 PMC3695748

[45] Chung J Kim KH Lee SC An SH Kwon K. Ursodeoxycholic acid (UDCA) exerts anti-atherogenic effects by inhibiting endoplasmic reticulum (ER) stress induced by disturbed flow. Mol Cells. (2015) 38(10):851–8. 10.14348/molcells.2015.0094 26442866 PMC4625066

[46] Canham L Sendac S Diagbouga MR Wolodimeroff E Pirri D Tardajos Ayllon B EVA1A (eva-1 homolog A) promotes endothelial apoptosis and inflammatory activation under disturbed flow via regulation of autophagy. Arterioscler Thromb Vasc Biol. (2023) 43(4):547–61. 10.1161/ATVBAHA.122.318110 36794585 PMC10026973

[47] Dong Y Zhang M Wang S Liang B Zhao Z Liu C Activation of AMP-activated protein kinase inhibits oxidized LDL-triggered endoplasmic reticulum stress in vivo. Diabetes. (2010) 59(6):1386–96. 10.2337/db09-1637 20299472 PMC2874699

[48] Tao YK Yu PL Bai YP Yan ST Zhao SP Zhang GQ. Role of PERK/eIF2α/CHOP endoplasmic reticulum stress pathway in oxidized low-density lipoprotein mediated induction of endothelial apoptosis. Biomed Environ Sci. (2016) 29(12):868–76. 10.3967/bes2016.116 28081747

[49] Hang L Peng Y Xiang R Li X Li Z. Ox-LDL causes endothelial cell injury through ASK1/NLRP3-mediated inflammasome activation via endoplasmic Reticulum stress. Drug Des Devel Ther. (2020) 14:731–44. 10.2147/DDDT.S231916 32158192 PMC7047838

[50] Wang X Gao F Cheng C Zhang Y. Knockdown of ADAMDEC1 ameliorates ox-LDL-induced endothelial cell injury and atherosclerosis progression. Funct Integr Genomics. (2023) 24(1):1. 10.1007/s10142-023-01278-8 38063920

[51] Irshad Z Xue M Ashour A Larkin JR Thornalley PJ Rabbani N. Activation of the unfolded protein response in high glucose treated endothelial cells is mediated by methylglyoxal. Sci Rep. (2019) 9(1):7889. 10.1038/s41598-019-44358-1 31133647 PMC6536510

[52] Outinen PA Sood SK Pfeifer SI Pamidi S Podor TJ Li J Homocysteine-induced endoplasmic reticulum stress and growth arrest leads to specific changes in gene expression in human vascular endothelial cells. Blood. (1999) 94(3):959–67. 10.1182/blood.V94.3.959.415k20_959_967 10419887

[53] Wang XC Sun WT Yu CM Pun SH Underwood MJ He GW ER stress mediates homocysteine-induced endothelial dysfunction: modulation of IKCa and SKCa channels. Atherosclerosis. (2015) 242(1):191–8. 10.1016/j.atherosclerosis.2015.07.021 26204495

[54] Murugan D Lau YS Lau CW Mustafa MR Huang Y. Angiotensin 1–7 protects against angiotensin II-induced endoplasmic reticulum stress and endothelial dysfunction via Mas receptor. PLoS One. (2015) 10(12):e0145413. 10.1371/journal.pone.0145413 26709511 PMC4692500

[55] Guo CL Liu HM Li B Lu ZY. Angiotensin-(1–9) prevents angiotensin II-induced endothelial apoptosis through CNPY2/PERK pathway. Apoptosis. (2023) 28(3-4):379–96. 10.1007/s10495-022-01793-2 36422742

[56] Hu HJ Jiang ZS Zhou SH Liu QM. Hydrogen sulfide suppresses angiotensin II-stimulated endothelin-1 generation and subsequent cytotoxicity-induced endoplasmic reticulum stress in endothelial cells via NF-κB. Mol Med Rep. (2016) 14(5):4729–40. 10.3892/mmr.2016.5827 27748925

[57] Gianopoulos I Daskalopoulou SS. Macrophage profiling in atherosclerosis: understanding the unstable plaque. Basic Res Cardiol. (2024) 119(1):35–56. 10.1007/s00395-023-01023-z 38244055

[58] Yao S Miao C Tian H Sang H Yang N Jiao P Endoplasmic reticulum stress promotes macrophage-derived foam cell formation by up-regulating cluster of differentiation 36 (CD36) expression. J Biol Chem. (2014) 289(7):4032–42. 10.1074/jbc.M113.524512 24366867 PMC3924270

[59] Kattoor AJ Goel A Mehta JL. LOX-1: regulation, signaling and its role in atherosclerosis. Antioxidants (Basel). (2019) 8(7):218. 10.3390/antiox8070218 31336709 PMC6680802

[60] Choromańska B Myśliwiec P Choromańska K Dadan J Chabowski A. The role of CD36 receptor in the pathogenesis of atherosclerosis. Adv Clin Exp Med. (2017) 26(4):717–22. 10.17219/acem/62325 28691408

[61] Devries-Seimon T Li Y Yao PM Stone E Wang Y Davis RJ Cholesterol-induced macrophage apoptosis requires ER stress pathways and engagement of the type A scavenger receptor. J Cell Biol. (2005) 171(1):61–73. 10.1083/jcb.200502078 16203857 PMC2171235

[62] Tabas I. The role of endoplasmic reticulum stress in the progression of atherosclerosis. Circ Res. (2010) 107(7):839–50. 10.1161/CIRCRESAHA.110.224766 20884885 PMC2951143

[63] Yao S Tian H Zhao L Li J Yang L Yue F Oxidized high density lipoprotein induces macrophage apoptosis via toll-like receptor 4-dependent CHOP pathway. J Lipid Res. (2017) 58(1):164–77. 10.1194/jlr.M071142 27895089 PMC5234716

[64] Guo C Ma R Liu X Chen T Li Y Yu Y Silica nanoparticles promote oxLDL-induced macrophage lipid accumulation and apoptosis via endoplasmic reticulum stress signaling. Sci Total Environ. (2018) 631-632:570–9. 10.1016/j.scitotenv.2018.02.312 29533793

[65] Li H Wang M Qu K Xu R Zhu H. MP allosterically activates AMPK to enhance ABCA1 stability by retarding the calpain-mediated degradation pathway. Int J Mol Sci. (2023) 24(24):17280. 10.3390/ijms242417280 PMC10743971 38139111

[66] Tabas I Ron D. Integrating the mechanisms of apoptosis induced by endoplasmic reticulum stress. Nat Cell Biol. (2011) 13(3):184–90. 10.1038/ncb0311-184 21364565 PMC3107571

[67] Feng B Yao PM Li Y Devlin CM Zhang D Harding HP The endoplasmic reticulum is the site of cholesterol-induced cytotoxicity in macrophages. Nat Cell Biol. (2003) 5(9):781–92. 10.1038/ncb1035 12907943

[68] Yao PM Tabas I. Free cholesterol loading of macrophages is associated with widespread mitochondrial dysfunction and activation of the mitochondrial apoptosis pathway. J Biol Chem. (2001) 276(45):42468–76. 10.1074/jbc.M101419200 11533046

[69] Li G Mongillo M Chin KT Harding H Ron D Marks AR Role of ERO1-alpha-mediated stimulation of inositol 1,4,5-triphosphate receptor activity in endoplasmic reticulum stress-induced apoptosis. J Cell Biol. (2009) 186(6):783–92. 10.1083/jcb.200904060 19752026 PMC2753154

[70] Timmins JM Ozcan L Seimon TA Li G Malagelada C Backs J Calcium/calmodulin-dependent protein kinase II links ER stress with Fas and mitochondrial apoptosis pathways. J Clin Invest. (2009) 119(10):2925–41. 10.1172/JCI38857 19741297 PMC2752072

[71] Hu H Tian M Ding C Yu S. The C/EBP homologous protein (CHOP) transcription factor functions in endoplasmic reticulum stress-induced apoptosis and microbial infection. Front Immunol. (2018) 9:3083. 10.3389/fimmu.2018.03083 30662442 PMC6328441

[72] Korbecki J Bajdak-Rusinek K. The effect of palmitic acid on inflammatory response in macrophages: an overview of molecular mechanisms. Inflammation Res. (2019) 68(11):915–32. 10.1007/s00011-019-01273-5 PMC6813288 31363792

[73] Shanahan CM Furmanik M. Endoplasmic reticulum stress in arterial smooth muscle cells: a novel regulator of vascular disease. Curr Cardiol Rev. (2017) 13(2):94–105. 10.2174/1573403X12666161014094738 27758694 PMC5440785

[74] Bennett MR Sinha S Owens GK. Vascular smooth muscle cells in atherosclerosis. Circ Res. (2016) 118(4):692–702. 10.1161/CIRCRESAHA.115.306361 26892967 PMC4762053

[75] Chattopadhyay A Kwartler CS Kaw K Li Y Kaw A Chen J Cholesterol-induced phenotypic modulation of smooth muscle cells to macrophage/fibroblast-like cells is driven by an unfolded protein response. Arterioscler Thromb Vasc Biol. (2021) 41(1):302–16. 10.1161/ATVBAHA.120.315164 33028096 PMC7752246

[76] Shankman LS Gomez D Cherepanova OA Salmon M Alencar GF Haskins RM KLF4-dependent phenotypic modulation of smooth muscle cells has a key role in atherosclerotic plaque pathogenesis. Nat Med. (2015) 21(6):628–37. 10.1038/nm.3866 25985364 PMC4552085

[77] Li Y Guo X Xue G Wang H Wang Y Wang W RNA splicing of the Abi1 gene by MBNL1 contributes to macrophage-like phenotype modulation of vascular smooth muscle cell during atherogenesis. Cell Prolif. (2021) 54(5):e13023. 10.1111/cpr.13023 33759281 PMC8088461

[78] Lin H Ni T Zhang J Meng L Gao F Pan S Knockdown of Herp alleviates hyperhomocysteinemia mediated atherosclerosis through the inhibition of vascular smooth muscle cell phenotype switching. Int J Cardiol. (2018) 269:242–9. 10.1016/j.ijcard.2018.07.043 30017525

[79] Hansson GK. Inflammation and atherosclerosis: the end of a controversy. Circulation. (2017) 136(20):1875–7. 10.1161/CIRCULATIONAHA.117.030484 28916641

[80] Long F Yang D Wang J Wang Q Ni T Wei G SMYD3-PARP16 axis accelerates unfolded protein response and mediates neointima formation. Acta Pharm Sin B. (2021) 11(5):1261–73. 10.1016/j.apsb.2020.12.010 34094832 PMC8148056

[81] Shemiakova T Ivanova E Wu WK Kirichenko TV Starodubova AV Orekhov AN. Atherosclerosis as mitochondriopathy: repositioning the disease to help finding new therapies. Front Cardiovasc Med. (2021) 8:660473. 10.3389/fcvm.2021.660473 34017868 PMC8129197

[82] Sorrentino V Menzies KJ Auwerx J. Repairing mitochondrial dysfunction in disease. Annu Rev Pharmacol Toxicol. (2018) 58:353–89. 10.1146/annurev-pharmtox-010716-104908 28961065

[83] Ma Y Sun W Ye Z Liu L Li M Shang J Oxidative stress biomarker triggered multiplexed tool for auxiliary diagnosis of atherosclerosis. Sci Adv. (2023) 9(41):eadh1037. 10.1126/sciadv.adh1037 37831761 PMC10575586

[84] Förstermann U Xia N Li H. Roles of vascular oxidative stress and nitric oxide in the pathogenesis of atherosclerosis. Circ Res. (2017) 120(4):713–35. 10.1161/CIRCRESAHA.116.309326 28209797

[85] Marchi S Patergnani S Pinton P. The endoplasmic reticulum-mitochondria connection: one touch, multiple functions. Biochim Biophys Acta. (2014) 1837(4):461–9. 10.1016/j.bbabio.2013.10.015 24211533

[86] Thoma A Lyon M Al-Shanti N Nye GA Cooper RG Lightfoot AP. Eukarion-134 attenuates endoplasmic reticulum stress-induced mitochondrial dysfunction in human skeletal muscle cells. Antioxidants (Basel). (2020) 9(8):710. 10.3390/antiox9080710 32764412 PMC7466046

[87] Win S Than TA Fernandez-Checa JC Kaplowitz N. JNK interaction with sab mediates ER stress induced inhibition of mitochondrial respiration and cell death. Cell Death Dis. (2014) 5(1):e989. 10.1038/cddis.2013.522 24407242 PMC4040675

[88] Liu C Zhang A. ROS-mediated PERK-eIF2α-ATF4 pathway plays an important role in arsenite-induced L-02 cells apoptosis via regulating CHOP-DR5 signaling. Environ Toxicol. (2020) 35(10):1100–13. 10.1002/tox.22946 32506763

[89] Santos CX Nabeebaccus AA Shah AM Camargo LL Filho SV Lopes LR. Endoplasmic reticulum stress and nox-mediated reactive oxygen species signaling in the peripheral vasculature: potential role in hypertension. Antioxid Redox Signaling. (2014) 20(1):121–34. 10.1089/ars.2013.5262 PMC3880927 23472786

[90] Xue X Piao JH Nakajima A Sakon-Komazawa S Kojima Y Mori K Tumor necrosis factor alpha (TNFalpha) induces the unfolded protein response (UPR) in a reactive oxygen species (ROS)-dependent fashion, and the UPR counteracts ROS accumulation by TNFalpha. J Biol Chem. (2005) 280(40):33917–25. 10.1074/jbc.M505818200 16107336

[91] Cao SS Kaufman RJ. Endoplasmic reticulum stress and oxidative stress in cell fate decision and human disease. Antioxid Redox Signaling. (2014) 21(3):396–413. 10.1089/ars.2014.5851 PMC4076992 24702237

[92] Wang J Toan S Zhou H. New insights into the role of mitochondria in cardiac microvascular ischemia/reperfusion injury. Angiogenesis. (2020) 23(3):299–314. 10.1007/s10456-020-09720-2 32246225

[93] Graham A Allen AM. Mitochondrial function and regulation of macrophage sterol metabolism and inflammatory responses. World J Cardiol. (2015) 7(5):277–86. 10.4330/wjc.v7.i5.277 26015858 PMC4438467

[94] Wang Y Wang GZ Rabinovitch PS Tabas I. Macrophage mitochondrial oxidative stress promotes atherosclerosis and nuclear factor-κB-mediated inflammation in macrophages. Circ Res. (2014) 114(3):421–33. 10.1161/CIRCRESAHA.114.302153 24297735 PMC3946745

[95] Pircher A Treps L Bodrug N Carmeliet P. Endothelial cell metabolism: a novel player in atherosclerosis? Basic principles and therapeutic opportunities. Atherosclerosis. (2016) 253:247–57. 10.1016/j.atherosclerosis.2016.08.011 27594537

[96] Chistiakov DA Shkurat TP Melnichenko AA Grechko AV Orekhov AN. The role of mitochondrial dysfunction in cardiovascular disease: a brief review. Ann Med. (2018) 50(2):121–7. 10.1080/07853890.2017.1417631 29237304

[97] Zalba G Fortuño A Orbe J San José G Moreno MU Belzunce M Phagocytic NADPH oxidase-dependent superoxide production stimulates matrix metalloproteinase-9: implications for human atherosclerosis. Arterioscler Thromb Vasc Biol. (2007) 27(3):587–93. 10.1161/01.ATV.0000256467.25384.c6 17194891

[98] Quesada IM Lucero A Amaya C Meijles DN Cifuentes ME Pagano PJ Selective inactivation of NADPH oxidase 2 causes regression of vascularization and the size and stability of atherosclerotic plaques. Atherosclerosis. (2015) 242(2):469–75. 10.1016/j.atherosclerosis.2015.08.011 26298737 PMC4818577

[99] Pandey VK Mathur A Khan MF Kakkar P. Activation of PERK-eIF2α-ATF4 pathway contributes to diabetic hepatotoxicity: attenuation of ER stress by Morin. Cell Signal. (2019) 59:41–52. 10.1016/j.cellsig.2019.03.008 30877037

[100] Liu C Yan DY Wang C Ma Z Deng Y Liu W IRE1 signaling pathway mediates protective autophagic response against manganese-induced neuronal apoptosis in vivo and in vitro. Sci Total Environ. (2020) 712:136480. 10.1016/j.scitotenv.2019.136480 31931206

[101] Kim YC Guan KL. mTOR: a pharmacologic target for autophagy regulation. J Clin Invest. (2015) 125(1):25–32. 10.1172/JCI73939 25654547 PMC4382265

[102] Song S Tan J Miao Y Zhang Q. Crosstalk of ER stress-mediated autophagy and ER-phagy: involvement of UPR and the core autophagy machinery. J Cell Physiol. (2018) 233(5):3867–74. 10.1002/jcp.26137 28777470

[103] Zhu L Wu G Yang X Jia X Li J Bai X Low density lipoprotein mimics insulin action on autophagy and glucose uptake in endothelial cells. Sci Rep. (2019) 9(1):3020. 10.1038/s41598-019-39559-7 30816192 PMC6395761

[104] Menghini R Casagrande V Marino A Marchetti V Cardellini M Stoehr R MiR-216a: a link between endothelial dysfunction and autophagy. Cell Death Dis. (2014) 5(1):e1029. 10.1038/cddis.2013.556 24481443 PMC4040670

[105] Lin L Zhang MX Zhang L Zhang D Li C Li YL. Autophagy, pyroptosis, and ferroptosis: new regulatory mechanisms for atherosclerosis. Front Cell Dev Biol. (2021) 9:809955. 10.3389/fcell.2021.809955 35096837 PMC8793783

[106] Tian H Zhang Z Han X Pan T Tao G Jiao P D4f alleviates the C/EBP homologous protein-mediated apoptosis in glycated high-density lipoprotein-treated macrophages by facilitating autophagy. Exp Biol Med. (2021) 246(24):2595–609. 10.1177/15353702211045323 PMC8669162 34525858

[107] Ge P Gao M Du J Yu J Zhang L. Downregulation of microRNA-512-3p enhances the viability and suppresses the apoptosis of vascular endothelial cells, alleviates autophagy and endoplasmic reticulum stress as well as represses atherosclerotic lesions in atherosclerosis by adjusting spliced/unspliced ratio of X-box binding protein 1 (XBP-1S/XBP-1U). Bioengineered. (2021) 12(2):12469–81. 10.1080/21655979.2021.2006862 34783632 PMC8810154

[108] Liu S Jiang X Cui X Wang J Liu S Li H Smooth muscle-specific HuR knockout induces defective autophagy and atherosclerosis. Cell Death Dis. (2021) 12(4):385. 10.1038/s41419-021-03671-2 33837179 PMC8035143

[109] Hui B Hou X Liu R Liu XH Hu Z. Gypenoside inhibits ox-LDL uptake and foam cell formation through enhancing Sirt1-FOXO1 mediated autophagy flux restoration. Life Sci. (2021) 264:118721. 10.1016/j.lfs.2020.118721 33160993

[110] Ozcan U Yilmaz E Ozcan L Furuhashi M Vaillancourt E Smith RO Chemical chaperones reduce ER stress and restore glucose homeostasis in a mouse model of type 2 diabetes. Science. (2006) 313(5790):1137–40. 10.1126/science.1128294 16931765 PMC4741373

[111] Lee B Rhead W Diaz GA Scharschmidt BF Mian A Shchelochkov O Phase 2 comparison of a novel ammonia scavenging agent with sodium phenylbutyrate in patients with urea cycle disorders: safety, pharmacokinetics and ammonia control. Mol Genet Metab. (2010) 100(3):221–8. 10.1016/j.ymgme.2010.03.014 20382058 PMC2905228

[112] Lynn EG Lhoták Š Lebeau P Byun JH Chen J Platko K 4-Phenylbutyrate protects against atherosclerotic lesion growth by increasing the expression of HSP25 in macrophages and in the circulation of Apoe(−/−) mice. FASEB J. (2019) 33(7):8406–22. 10.1096/fj.201802293RR 30964709

[113] Zhu G Gao H Li Y Li X Yang X Wang C Suppression of endoplasmic reticulum stress by 4-PBA enhanced atherosclerotic plaque stability via up-regulating CLOCK expression. Pathol Res Pract. (2024) 253:154969. 10.1016/j.prp.2023.154969 38029715

[114] Dong Y Zhang M Liang B Xie Z Zhao Z Asfa S Reduction of AMP-activated protein kinase alpha2 increases endoplasmic reticulum stress and atherosclerosis in vivo. Circulation. (2010) 121(6):792–803. 10.1161/CIRCULATIONAHA.109.900928 20124121 PMC2825900

[115] Engin F Hotamisligil GS. Restoring endoplasmic reticulum function by chemical chaperones: an emerging therapeutic approach for metabolic diseases. Diabetes Obes Metab. (2010) 12(Suppl 2):108–15. 10.1111/j.1463-1326.2010.01282.x 21029307

[116] Wang X Zhang Y Du L Jiang Z Guo Y Wang K TUDCA alleviates atherosclerosis by inhibiting AIM2 inflammasome and enhancing cholesterol efflux capacity in macrophage. iScience. (2024) 27(6):109849. 10.1016/j.isci.2024.109849 38784008 PMC11112614

[117] Yildirim Z Baboo S Hamid SM Dogan AE Tufanli O Robichaud S Intercepting IRE1 kinase-FMRP signaling prevents atherosclerosis progression. EMBO Mol Med. (2022) 14(4):e15344. 10.15252/emmm.202115344 35191199 PMC8988208

[118] Prola A Pires Da Silva J Guilbert A Lecru L Piquereau J Ribeiro M SIRT1 protects the heart from ER stress-induced cell death through eIF2α deacetylation. Cell Death Differ. (2017) 24(2):343–56. 10.1038/cdd.2016.138 27911441 PMC5299716

[119] Zheng G Li H Zhang T Yang L Yao S Chen S Irisin protects macrophages from oxidized low density lipoprotein-induced apoptosis by inhibiting the endoplasmic reticulum stress pathway. Saudi J Biol Sci. (2018) 25(5):849–57. 10.1016/j.sjbs.2017.08.018 30108431 PMC6087810

[120] Qiu J Fu Y Chen Z Zhang L Li L Liang D BTK Promotes atherosclerosis by regulating oxidative stress, mitochondrial injury, and ER stress of macrophages. Oxid Med Cell Longevity. (2021) 2021:9972413. 10.1155/2021/9972413 PMC8175170 34136067

[121] Wei Q Ren H Zhang J Yao W Zhao B Miao J. An inhibitor of Grp94 inhibits OxLDL-induced autophagy and apoptosis in VECs and stabilized atherosclerotic plaques. Front Cardiovasc Med. (2021) 8:757591. 10.3389/fcvm.2021.757591 34938782 PMC8687133

[122] Cheng CK Luo JY Lau CW Cho WC Ng CF Ma RCW A GLP-1 analog lowers ER stress and enhances protein folding to ameliorate homocysteine-induced endothelial dysfunction. Acta Pharmacol Sin. (2021) 42(10):1598–609. 10.1038/s41401-020-00589-x 33495519 PMC8463564

[123] Xiong W Fei M Wu C Wang W Luo R Shen L Atorvastatin inhibits endoplasmic reticulum stress through AMPK signaling pathway in atherosclerosis in mice. Exp Ther Med. (2020) 19(3):2266–72. 10.3892/etm.2019.8379 32104293 PMC7027330

[124] Cheang WS Ngai CY Tam YY Tian XY Wong WT Zhang Y Black tea protects against hypertension-associated endothelial dysfunction through alleviation of endoplasmic reticulum stress. Sci Rep. (2015) 5:10340. 10.1038/srep10340 25976123 PMC4432571

[125] Song J Li J Hou F Wang X Liu B. Mangiferin inhibits endoplasmic reticulum stress-associated thioredoxin-interacting protein/NLRP3 inflammasome activation with regulation of AMPK in endothelial cells. Metabolism. (2015) 64(3):428–37. 10.1016/j.metabol.2014.11.008 25499441

[126] Choi SK Lim M Byeon SH Lee YH. Inhibition of endoplasmic reticulum stress improves coronary artery function in the spontaneously hypertensive rats. Sci Rep. (2016) 6:31925. 10.1038/srep31925 27550383 PMC4994042

